# Evaluation of Sulfated Polysaccharides from the Brown Seaweed *Dictyopteris Justii* as Antioxidant Agents and as Inhibitors of the Formation of Calcium Oxalate Crystals

**DOI:** 10.3390/molecules181214543

**Published:** 2013-11-25

**Authors:** Karoline Rachel Teodosio Melo, Rafael Barros Gomes Camara, Moacir Fernandes Queiroz, Arthur Anthunes Jacome Vidal, Camila Renata Machado Lima, Raniere Fagundes Melo-Silveira, Jailma Almeida-Lima, Hugo Alexandre Oliveira Rocha

**Affiliations:** 1 Laboratório de Biotecnologia de Polímeros Naturais (BIOPOL), Departamento de Bioquímica, Centro de Biociências, Universidade Federal do Rio Grande do Norte (UFRN), Natal, Rio Grande do Norte 59072-970, Brazil; E-Mails: melo.krt@gmail.com (K.R.T.M.); rafael_bgc@yahoo.com.br (R.B.G.C.); moacirfqn@gmail.com (M.F.Q.); arthur_bio@hotmail.com (A.A.J.V.); ranierefagundes@hotmail.com (R.F.M.-S.); biolottus23@yahoo.com.br (J.A.-L.); 2 Instituto de Química, Universidade Federal do Rio Grande do Norte (UFRN), Natal, Rio Grande do Norte 59072-970, Brazil; E-Mail: camilarmdelima@gmail.com

**Keywords:** fucan, glucan, urolithiasis, bioactive polysaccharides

## Abstract

Oxalate crystals and other types of crystals are the cause of urolithiasis, and these are related to oxidative stress. The search for new compounds with antioxidant qualities and inhibitors of these crystal formations is therefore necessary. In this study, we extracted four sulfated polysaccharides, a fucoglucoxyloglucuronan (DJ-0.3v), a heterofucan (DJ-0.4v), and two glucans (DJ-0.5v and DJ-1.2v), from the marine alga *Dictyopteris justii*. The presence of sulfated polysaccharides was confirmed by chemical analysis and FT-IR. All the sulfated polysaccharides presented antioxidant activity under different conditions in some of the *in vitro* tests and inhibited the formation of calcium oxalate crystals. Fucan DJ-0.4v was the polysaccharide that showed the best antioxidant activity and was one of the best inhibitors of the crystallization of calcium oxalate. Glucan DJ-0.5v was the second most potent inhibitor of the formation of oxalate crystals, as it stabilized dehydrated oxalate crystals (less aggressive form), preventing them from transforming into monohydrate crystals (more aggressive form). The obtained data lead us to propose that these sulfated polysaccharides are promising agents for use in the treatment of urolithiasis.

## 1. Introduction

The marine environment provides endless supplies of compounds that offer human health benefits such as fatty acids, minerals, vitamins, and bioactive peptides [1,2]. Among marine organisms, seaweeds have aroused special interest as a good source of several components with biotechnological potential [3]. When thinking of important activities for the food industry, cosmetics, and medicines, one of the algal compounds that stand out are the sulfated polysaccharides [4].

Brown seaweeds mostly synthesize a family of polydisperse polysaccharides composed of sulfated L-fucose (sulfated homo- and heterofucans) [5], yet other sulfated heteropolysaccharides have been found in these algae too. These polysaccharides have been described as having various activities such as antithrombotic [6], antinociceptive [7], anti-inflammatory [8], antiviral, antitumor [9], and antioxidant properties [10].

With the advancement of the study of chronic diseases and their prevention, emphasis has been given to the search for antioxidant molecules. According to Valko *et al.* [11], an ideal antioxidant should be able to remove free radicals, chelate transition metals, interact with other antioxidants, and be absorbed, besides working both in aqueous solutions and in areas of the cell membrane (lipophilic environment). However, the substances discovered up to now which act as antioxidants, usually have only one or some of those characteristics [11].

The mechanisms of the antioxidant activities of the sulfated polysaccharides of brown algae are quite diverse. Previous studies have reported the prevention of lipid peroxidation and the capacity to abduct reactive species—such as the hydroxyl radical and superoxide anion—passing through the chelation of metal ions [12], and including the prevention of the formation of reactive species. These antioxidant properties may be important for the treatment of various diseases such as cancer and atherosclerosis, as well as for protection against tissue damage caused by oxidants. For example, it has been reported that high concentrations of calcium oxalate lead to the production of reactive oxygen species (ROS) in tissue cultures as in *in vivo* models [13], and it has been demonstrated that homofucans extracted from the seaweed *Fucus vesiculosus* can protect kidney tissues from the damage caused by oxidative stress resulting from the presence of oxalate [14].

Crystal-forming oxalate salts go through two physico-chemical phases: nucleation and aggregation. The crystal growth, which could be considered a third phase, also occurs. It has been suggested that ROS increase the amount of oxalate crystals since they modulate the process of nucleation, growth and crystal aggregation [15] and it has been confirmed that antioxidants such as vitamin E and ascorbic acid promote the reduction in the size of oxalate crystals and the resulting kidney injuries caused by them [16]. Therefore, sulfated polysaccharides could protect the renal tissue from the aggression caused by oxalate, and by another mechanism they could inhibit the formation of oxalate crystals, as demonstrated by Zhang and colleagues [17]. These authors showed that polysaccharides from the brown seaweed *Sargassum graminifolium* were able to inhibit the crystallization of calcium oxalate *in vitro*.

Oxalate crystals and other types of crystal are the causative agents of urinary lithiasis, or urolithiasis. This disease affects some 10% of the world population and 60%–90% of the cases are caused primarily by calcium oxalate crystals [18]. The literature also shows that the probability of recurrence of these crystal formations is more than 60%, and despite advances in medical treatments, there are currently no satisfactory drugs for the treatment of urolithiasis [19]. Therefore, a search is underway for sources of molecules that can provide effective treatment of urolithiasis.

Several species of brown seaweed can be found along the Brazilian northeastern coast, but none of the sulfated polysaccharides in these seaweeds has been evaluated as an inhibitor of calcium oxalate crystal formation. Accordingly, the aim of this work was to assess the antioxidant activity of sulfated polysaccharides from the brown seaweed *Dictyopteris justii*, found in large quantities, and its effect on the crystallization of calcium oxalate *in vitro*.

## 2. Results and Discussion

### 2.1. Chemical Analysis of Sulfated Polysaccharides Obtained

Sulfated polysaccharides (SPs) of *D. justii* were solubilized in the presence of proteolytic enzymes, which degraded the contaminating proteins. Subsequently, they were separated into four fractions with the use of differential precipitation with acetone. These fractions were termed DJ-0.3v, DJ-0.4v, DJ-0.5v, and DJ-1.2v, and subjected to the analyses described below.

Table 1 shows the summary of data obtained from chemical analysis. From the results, the presence of sugars can be observed in all fractions, ranging from 80.4% to 59.6%. These values can be considered high when compared with the values found in other sulfated polysaccharides of brown seaweed such as *Spatoglossum schröederi* [20] and *Canistrocarpus cervicornis* [5], which were no higher than 50%, indicating that the sugar content varies according to the species of the studied algae.

**Table 1:** Chemical analysis and molar ratio of the sugar and sulfate content of Sulfated Polysaccharides extracted from the Brown Seaweed *Dictyopteris justii.*

SPs	Total Sugar (%)	Sulfate (%)	Protein (%)	Molar Ratio ^1^				
				Glu ^1^	Xil ^1^	Glu. Ac. ^1^	Fuc ^1^	Sulfate
DJ-0.3v	67.5 ± 0.8	3.9 ± 0.4	1.6 ± 0.05	1.0	0.8	1.2	0.3	0.9
DJ-0.4v	59.6 ± 1.3	7.5 ± 1.8	0.9 ± 0.04	1.0	1.7	1.4	1.2	2.1
DJ-0.5v	75.8 ± 0.1	4.3 ± 0.6	0.1 ± 0.02	1.0	0.0	0.0	0.2	1.0
DJ-1.2v	80.4 ± 0.2	6.8 ± 0.2	n.d	1.0	0.0	0.0	0.1	1.5

**^1^** Molar ratio of sugars and sulfate using glucose as reference; n.d–Not detected; Glu–Glucose; Xil–Xylose; Fuc–Fucose; Glu. Ac.–Glucuronic Acid.

The sulfate content of the fractions, on the other hand, showed a variation of 3.9% to 7.5%, being the highest percentage found in the fraction DJ-0.4v. When comparing the values recorded in the sulfate content of the polysaccharide *D. justii* with those described for the sulfated polysaccharides of *Dictyopteris delicatula*—also collected in the same region—it is clear that the percentage of sulfate of the *D. justii* SPs presents a lower value than the polysaccharides of the *D. delicatula* seaweed since the latter’s range was from 14% to 19% [21]. However, another recent study, conducted by Camara and colleagues [5], using the brown seaweed *C. cervicornis*, has shown fucans with sulfate levels around 2.8%. Thus, it is clear that the amount of sulfate and total sugar content of seaweed may vary between species of the same genus. As for contamination by proteins, the range was low, ranging from 0% to 1.6%, respectively.

Referring to the monosaccharide composition, also shown in Table 1, it can be observed that the sulfated polysaccharides of the obtained fractions from *D. justii* are heterogeneous polymers. From the data it could be concluded that glucose and fucose are the monosaccharides present in all fractions; however, the quantity of these sugars is different in each polymer, making it clear that the percentages of these sugars may vary according to the extracted polysaccharide. Furthermore, it can be clearly noticed that the alga *D. justii* synthesizes different populations of sulfated polysaccharides*.* The first one is DJ-0.3v, which is rich in glucose, xylose, and glucuronic acid and shows traces of fucose; the second one is DJ-0.4v, with differentiating high amounts of fucose. The two populations designated by DJ-0.5v and DJ-1.2v simply show glucose and traces of fucose, but differ from each other in the amount of sulfate ions.

Thus, it can be inferred that the alga *D. justii* synthesizes a glucufucoxyloglucuronan (DJ-0.3v) and a heterofucan (DJ-0.4V). A great number of studies have shown that brown seaweed synthesize more than one type of fucan, for example, *Laminaria japonica* [22], and species of the order Dictyotales such as *Dictyota menstrualis* [7], *C. cervicornis* [5], and *D. delicatula* [21]. There have also been reports of the presence of sulfated polysaccharides in brown algae which do not have fucose as the main component, as observed in algae *Sargassum stenophyllum* [23]. Thus, the profile was as expected: the presence of heteropolymers is very common in brown algae [24].

Surprisingly, the presence of high concentrations of glucose as well as sulfate was observed in fractions DJ-0.5v and DJ-1.2v, indicating the presence of sulfated glucans. Glucose is not very common in the composition of heterofucans, but it has already been detected as part of the constitution of fucans from some seaweeds such as *D. cervicornis* [5], *Padina pavonia*, *S. stenophyllum,* and *Chorda filum* [25]. Nevertheless, no studies were found that demonstrate brown seaweed synthesizing sulfated glucans, thus, this is the first report of the presence of these polysaccharides in brown algae.

In order to determine the structural characteristics of the sulfated polysaccharides DJ-0.3v and DJ-0.4v, and of the sulfated glucans DJ-0.5v and DJ-1.2, the samples were subjected to infrared spectroscopy and the results are shown in Table 2.

**Table 2:** IR spectra data of sulfated polysaccharides from the brown seaweed *D. justii.*

Sulfated Polysaccharides	IR (KBr) (cm^−1^)
DJ-0.3v	3,303, 2,924, 1,605, 1,231, 1,159, 1,024, 813
DJ-0.4v	3,337, 2,920, 1,603, 1,230, 1,162, 1,031, 811
DJ-0.5v	3,316, 2,924, 1,625, 1,244, 1,155, 1,033, 889
DJ-1.2v	3,324, 2,921, 1,630, 1,244, 1,158, 1,025, 994, 887

The bands in the region of 3,324–3,303 cm^−1^and 2,920–2,930 cm^−^^1^ demonstrate that the samples are polysaccharides, as they indicate the presence of OH and C-H groups, respectively, found in all polysaccharides. The band in the region around 1,605 cm^−1^ shows the presence of the carboxyl groups from the glucuronic acid [26] and it was only found in DJ-0.3v and DJ-0.4V. Since this peak signal presents itself in the form of a broad signal, a superimposition occurred over the signal of the solvation layer of the water, which is why the signal corresponding to bound water was not detected in the spectra of both SPs. On the other hand, the bands of the hydroxyls of the bound water in the spectra of the glucans have been identified in the region of 1,630–1,625 cm^−1^, since these do not present glucuronic acid in their composition. The asymmetric vibration of the C-O-C glycosidic bond appears in all spectra near the region of 1,150 cm^−^^1^, indicating the presence of pyranosidic rings in all the studied carbohydrates [27]. The bands around 1,232–1,256 cm^−1^ indicate an asymmetric S=O vibration [22] and the bands around 1,150 and 1,025–1,033 cm^−1^ indicate vibrations associated with C-O-S=O grouping [28], confirming the presence of sulfated polysaccharides in all samples. The bands observed in 813 cm^−1^ (DJ-0.3V) and 811 cm^−^^1^ (DJ-0.4V) indicate the presence of sulfate predominant at position 6 of the sugar residues of these polymers [29]. On the other hand, the bands at 889–887 cm^−^^1^ indicate the presence of sulfate at position 6 of glucose residues [30].

### 2.2. Antioxidant Activity

Seaweeds inhabit midcoastal areas, especially in harsher environments where they are subjected to repeated immersions and emersions due to tidal fluctuations. As a result, it is exposed twice a day to a variety of environmental stresses, including exposure to ultraviolet radiation, rapid temperature fluctuations, osmotic stress, and desiccation [31,32]. Some of these factors contribute to the generation of free radicals, which in most cases are highly reactive and, therefore, cause damage to the cell structures of algae. However, there are no records of great damage caused by these agents in seaweed, which indicates that there exists a defense mechanism mediated by an efficient antioxidant system constituted of enzymes and probably a myriad of antioxidant molecules. Among these molecules, several studies have been highlighting sulfated polysaccharides as potent antioxidants [3,10,12].

The term antioxidant refers to compounds that can prevent the formation of biological substances and chemical oxidation damage induced by reactive species. The formation process of these reactive species occurs through a chain reaction involving three stages—initiation, propagation and termination—wherein the antioxidants act through several mechanisms. Thus, different methods are used to evaluate the effect of sulfated polysaccharides of *D. justii* in the different stages of initiation (total antioxidant capacity and reducing power), propagation (chelation of copper and iron), and termination (sequestration of the superoxide and hydroxyl radicals).

#### 2.2.1. TAC (Total Antioxidant Capacity)

**Figure 1:**
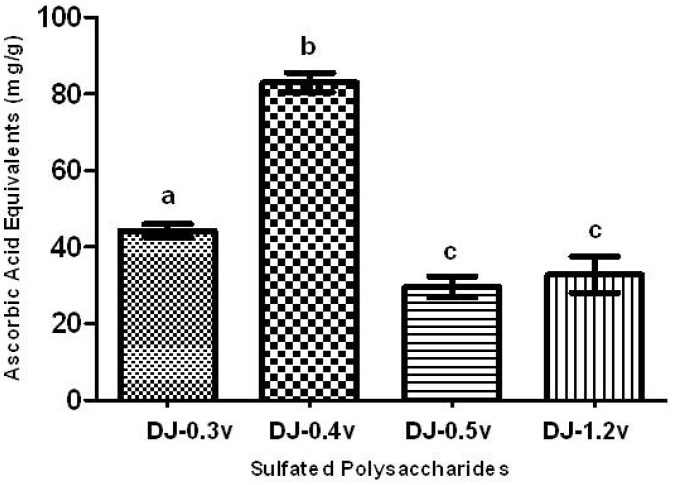
Total antioxidant capacity of sulfated polysaccharides extracted from the marine brown seaweed *D. justii*. The results are expressed as AAE. Each value is the mean ± SD of three determinations: Different letters (**a**, **b**, **c**) indicate a significant difference (*p* < 0.05) between sulfated polysaccharides.

The TAC test aims to evaluate the ability of a sample to donate electrons, thus neutralizing compounds such as free radicals, like Reactive Oxygen Species (ROS). The results are presented in the form of ascorbic acid equivalents (AAE), or, in other words, mg of ascorbic acid/g of extract (Figure 1).

All SPs presented activity in the TAC assay, as shown in Figure 1. The polysaccharide that showed a significantly higher TAC (*p* < 0.05) was fucan DJ-0.4v, which demonstrated a value of 82.9 mg/g of AAE, while DJ-0.3v had a TAC value of 44.3 mg/g. The TAC values for the glucans DJ-0.5v and DJ-1.2v, 29.6 and 32.8 mg/g of AAE, respectively, were significantly lower than those found for other polysaccharides. Furthermore, when they were compared with each other, there was no significant difference between them. In contrast, the fucans showed better results than those found for the fucans from the alga *C. cervicornis* [5]; the fucans of this alga showed values of TAC ranging between 20.9 and 39.4 mg/g of AAE. Thus, the detected values here are extremely interesting, which prompted us to conduct further antioxidant tests to determine the potential antioxidant mechanisms of the sulfated polysaccharides of *D. justii*.

#### 2.2.2. Reducing Power

**Figure 2:**
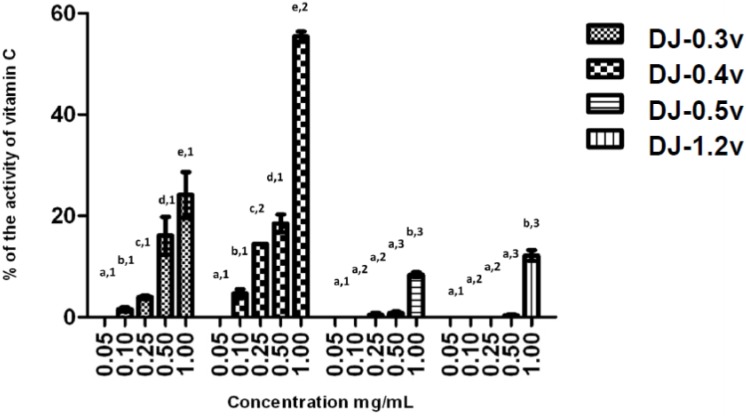
Reducing power of sulfated polysaccharides from *D. justii*. Data are expressed as means ± standard deviation. Reducing power is expressed as a percentage of the activity shown by 0.2 mg/mL of ascorbic acid. Different letters (**a**, **b**, **c**, **d**, **e**) indicate a significant difference between concentrations of individual algal sulfated polysaccharides by one-way ANOVA followed by Student-Newman-Keuls test (*p* < 0.05). Different numbers (1, 2, 3, 4) indicate a significant difference (*p* < 0.05) between the same concentrations of different sulfated polysaccharides.

The reducing power test evaluates the capacity of a sample to donate electrons. The result of this test is expressed in reducing activity equivalent to ascorbic acid in a concentration of 0.1 mg/mL (Figure 2). The data obtained from the test of the reducing power present a profile similar to that observed in the TAC test; in other words, DJ-0.3v and DJ-0.4v were more potent than the glucans. DJ-0.3V exhibited a dose-dependent effect, but upon comparing its activity with that of the fucan DJ-0.4v, it was observed that DJ-0.3v had lower reducing agent potency. This fact was observed at all tested concentrations and it was even more evident at the highest evaluated concentration (1.0 mg/mL) because at this concentration, DJ-0.4v presented twice the reducing power capacity as that observed in DJ-0.3v. The values obtained with DJ-0.4v were close to those obtained with a fucan extracted from *D. delicatula*, another alga of the genus *Dictyopteris*. This fucan, called F1.3v, presented an activity of 53.2% of vitamin C activity [21]. The glucans DJ-0.5v and DJ-1.2v were less potent and presented a maximum activity of 8.8% and 12.2% (1.0 mg/mL), respectively.

The reducing effect of the compounds, including sulfated polysaccharides, seems to function as an inhibitor of chain reactions of free radicals by means of donation of electrons, since this activity is mediated by redox reactions [33]. Zhang and colleagues [34] reported that the density of negative charges in a fucan is important for it to be a good electron donor and therefore presents a good reducing power. This is also valid for glucans, as Telles and colleagues have already shown [35]. These authors have promoted the sulfation of a glucan and verified that the insertion of sulfate groups increased the reducing power of glucans. However, the literature supports the observation that only the density of negative charges itself is not sufficient to justify a higher reducing power of a polysaccharide; the pattern of distribution of charges in the molecule also appears to be an important factor so that the polysaccharide can present reducing power. This justifies the fact that DJ-0.4v presents reducing power similar to that of fucan F1.3v of the alga *D. delicatula* [21] although DJ-0.4v is less sulfated than fucan F1.3v.

In living organisms, the intracellular environment is different from the extracellular environment. Furthermore, the organelles within the cells have different environmental conditions, for example, lysosome and mitochondria. Thus, we used two methods that evaluate the ability/capacity of a sample to donate electrons because we simulate situations that may be encountered in living organisms. Since the chemical environment of each method is different, a molecule can exhibit good activity in one method but not in the other. Our data showed that sulfated polysaccharides from *D. justii* are good donors of electrons under different conditions.

#### 2.2.3. Hydroxyl and Superoxide Radical Scavenging

Hydroxyl radicals and superoxide anions are reactive oxygen species (ROS) implicated in cell damage. The hydroxyl radical is the most reactive of the radicals, making it extremely harmful. Its main source of production *in vivo* is due to the reaction of transition metals with the superoxide ion by the Fenton reaction [11]. On the other hand, superoxide anion is considered a primary ROS, capable of generating reactive derivatives by direct interaction with other molecules or by means of processes catalyzed by metals or enzymes [11] being also produced within the mitochondria. Due to the harmful effect in the body, these ROS are associated with numerous diseases, such as strokes, cancer, diabetes, liver, and neuronal lesions [36].

No polysaccharide of the *D. justii* alga presented hydroxyl scavenging activity and only DJ-0.4v (0.25 mg/mL) showed superoxide ion scavenging activity (29.4%). The absence or presence of low activity in the test of elimination of superoxide and hydroxyl radicals is common in sulfated polysaccharides extracted from brown algae [10]. This shows that the elimination of hydroxyl radicals is probably not the main antioxidant mechanism of these polysaccharides.

#### 2.2.4. Iron Chelating Ability

The chelating effect is very important since it inhibits the interaction between lipids and metals by forming insoluble metal complexes with ferrous ions. Furthermore, it is an effective way to eliminate the generation of hydroxyl radicals since it prevents iron from interacting with H_2_O_2_, thus preventing the decomposition of H_2_O_2 _and the formation of an even more damaging free radical. Of the sulfated polysaccharides extracted from *D. justii*, only DJ-0.3v and DJ-0.4v were capable of chelating iron; however, this activity was only moderate, reaching 23.7% and 27.6%, respectively. On the other hand, the glucans have shown no ferric chelating activity despite containing sulfate in their composition. Some authors correlate the ability of chelation of ferric sulfate polysaccharide to the presence of polymers. In fact, there are examples of polysaccharides without iron chelating activity that, when sulfated artificially, begin presenting this activity [37,38]. However, studies by Telles *et al.* [35] showed that the sulfation of glucans improved antioxidant activity in different assays. However, the sulfated glucan did not present significantly different iron chelation from the non-sulfated glucan, thus corroborating the hypothesis presented here, that it is not only the presence of sulfate that allows chelating as well as antioxidant activity of a polysaccharide but also the way these sulfate groups are distributed in the molecule.

#### 2.2.5. Copper Chelating Ability

**Figure 3:**
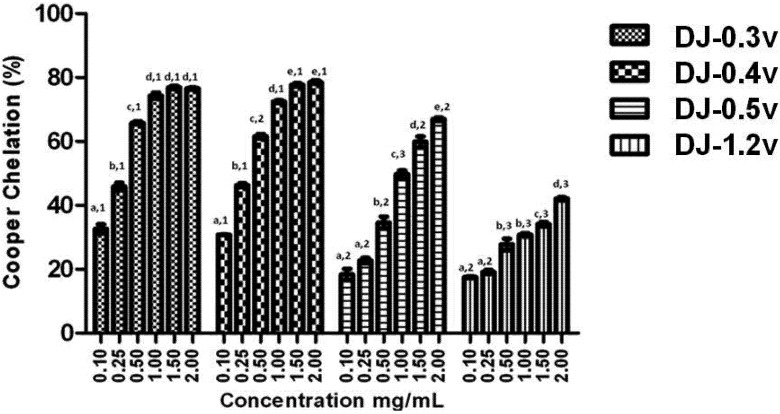
Copper chelating activity of sulfated polysaccharides from the brown algae (seaweed) *D. justii*. Each value is the mean ± standard deviation of three determinations: Different letters (**a**, **b**, **c**, **d**, **e**) indicate a significant difference (*p* < 0.05) between each concentration of the same sulfated polysaccharide. Different numbers (1, 2, 3) indicate a significant difference (*p* < 0.05) between the same concentrations of different sulfated polysaccharides.

The equilibrium of the concentration of copper ions in biological systems is crucial for the regulation of cellular functions. When an imbalance occurs in its concentration in the human body, this could lead to the development of severe health conditions such as osteoporosis, hypothyroidism, schizophrenia, premenstrual syndrome, *etc*. [39]. When this imbalance is caused by the increase in the concentration of copper, there is an increase in the production of reactive oxygen species, due in large part to Fenton [40] and Haber-Weiss [41] reactions. In addition, through the Fenton reaction, the preformed lipid hydroperoxides (LOOH) are decomposed to form alkoxyl radicals (LO), strong oxidizing agents, which can propagate the chain reaction of lipid peroxidation [41] or react with other cellular constituents. Consequently, the chelation of Cu^2+^ ions may be crucial for the prevention of the production of reactive species that damage the target biomolecules. Therefore, we have verified the chelating effect of copper ions, displayed by different concentrations of the fractions obtained from *D. justii* (Figure 3).

It can be observed from the data presented in Figure 3 that all polysaccharides have copper chelating activity and in all cases the effect is dose dependent. It is worth noting that once again DJ-0.3v and DJ-0.4v were more potent than the glucans. However, this time DJ-0.3v was more potent than DJ-0.5v, for it reached the plateau of its activity at a concentration lower than that of DJ-0.5v. Studies with fucans from the *Undaria pinnatifida* [42] alga have shown they also have the capacity to chelate copper and those with higher sulfate content have a higher activity, like the fucans of *D. justii*. We were unable to identify any other studies that evaluated the effect of fucans or glucans of other species such as copper chelators. Therefore, there is not yet enough data to make further observations on the activity of these polysaccharides.

### 2.3. *In Vitro* Assay of Calcium Oxalate Salts Crystallization Inhibition

Recent research has reported that the supersaturation of urine by calcium oxalate is primarily responsible for the formation of kidney stones. In this context, two separate processes are involved—nucleation and aggregation (clustering). When urinary supersaturation promoted by calcium oxalate exceeds the limit of metastability, oxalate ions and calcium cluster together and start to form the core of the crystal, which does not yet have the geometric shape of a crystal (nucleation). With the addition of new ions, this begins to grow (growth) in an orderly manner, generating a nanocrystal. Consequently, the growing nanocrystals aggregate with one to another, forming clusters (aggregation clustering) [43].

With therapeutic intent, seaweeds have been widely used in Eastern medicine to treat and/or prevent the damage caused by the formation of calcium oxalate crystals in the urinary tract [44]. In this search, from a salt mixture that simulates supersaturated urine, we checked the inhibitory effect of antioxidant sulfate polysaccharides of *D. justii* on the crystallization of calcium oxalate (CaOx), based on linear regression calculations as described in The Experimental section. It is noteworthy that this technique is not able to evaluate the process of nucleation and growth separately. However, it is widely used in studies found on this topic.

**Figure 4:**
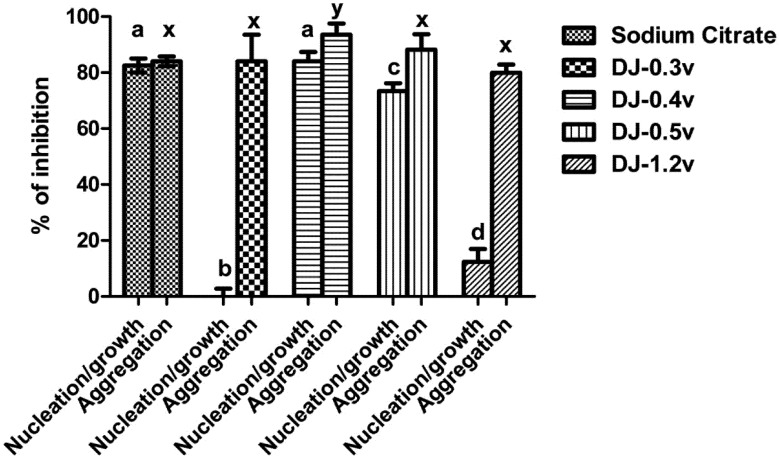
Inhibition crystallization of salts of calcium oxalate activity of sulfated polysaccharides from the brown seaweed *D. justii*. Different letters (**a**, **b**, **c**, **d**, **x**) indicate a significant difference (*p* < 0.05) between 0.25 mM of sodium citrate and different polysaccharides.

In Figure 4 it can be observed that the fraction DJ-0.3v was not able to inhibit nucleation and DJ-1.2v had low activity, with only 12.4% of inhibition. The best results were those observed for the polysaccharides DJ-0.5v and DJ-0.4v, whose nucleation inhibition capacity was 73.33% and 84.07%, respectively, a similar effect to that observed with sodium citrate (0.25 mM). Regarding the clustering of the crystals, the values of aggregation inhibition found in the presence of polysaccharides varied between 86% and 80%. Only the values of DJ-0.4v were statistically different from other polysaccharides (86%), and even greater than sodium citrate, whose clustering inhibition capacity was 84%.

The nucleation/growth and clustering of oxalate crystals can be inhibited by the presence of polyanions such as proteins and carbohydrates. This is primarily due to the presence of the negative charges of these molecules since these molecules interact with calcium and decrease the super-saturation system [45], which justifies our results with DJ-0.5v and DJ-0.4v. However, DJ-0.3v did not affect the nucleation and DJ-1.2v had very little effect, even though they are polyanions. A study of different glycosaminoglycans (animal sulfated polysaccharides) showed that each of them inhibits nucleation by a mechanism different from that used by the other glycosaminoglycans. It has been proposed that the inhibition of nucleation by sulfated polysaccharides is not only an effect of charges, but also how these charges are distributed in the molecule [46]. This would explain in part the effects of DJ-0.3v and DJ-1.2v.

Furthermore, it must be borne in mind that the nucleation/growth of the nanocrystals also depends on the relationship between the crystal size and the molecular weight as well as the volume of the conformation assumed by the polyanion in solution—the larger the size of the compound the less it interferes with the nucleation [47]. Therefore, we evaluated whether these parameters would also explain the lower inhibitory effect of nucleation met with DJ-0.3v and DJ-1.2v. The molecular weight of DJ-0.3v was about 30 kDa, whereas the other polysaccharides showed a molecular weight of about 16 kDa. In addition, the obtained values of the polysaccharide conformation volumes showed that DJ-0.3v had a size (~15 nm) much larger than that of the other polysaccharides (~2 nm) in solution. These data would explain why DJ-0.3v had the smallest inhibitory effect.

Other factors that may affect nucleation/growth are the topology of the crystal and interaction between the polyanion and the faces of the crystal. The crystals have different faces, one rich in Ca^2+^ and the other rich in oxalate. The polysaccharide, to be a good inhibitor of nucleation/growth, must be able to interact with the face rich in Ca^2+^, and for such a conformation that the polysaccharide assumes it is an important factor [47]. Therefore, we suggest that DJ-1.2v assumes a conformation that does not allow itself a good interaction with the Ca^2+^ face of the nascent crystals, leaving it ineffective as an inhibitor of nucleation.

The results with sulfated polysaccharides from *D. justii* led to the observation that some of the polysaccharides of this seaweed have a capacity similar to that of sodium citrate to strongly inhibit the formation of oxalate crystals. Furthermore, the values obtained here are better than those reported for the sulfated polysaccharides of brown seaweed *Sargassum graminifolium* [17], in which, in this case, the values of nucleation and aggregation inhibition obtained with polysaccharides of *S. graminifolium* did not exceed 70% and 77%, respectively. By these data, we sought to verify possible changes in the morphology of the crystals formed after treatment with sulfated polysaccharides extracted from *D. justii.* These results are expressed below.

### 2.4. Effect on Crystal Morphology

**Figure 5:**
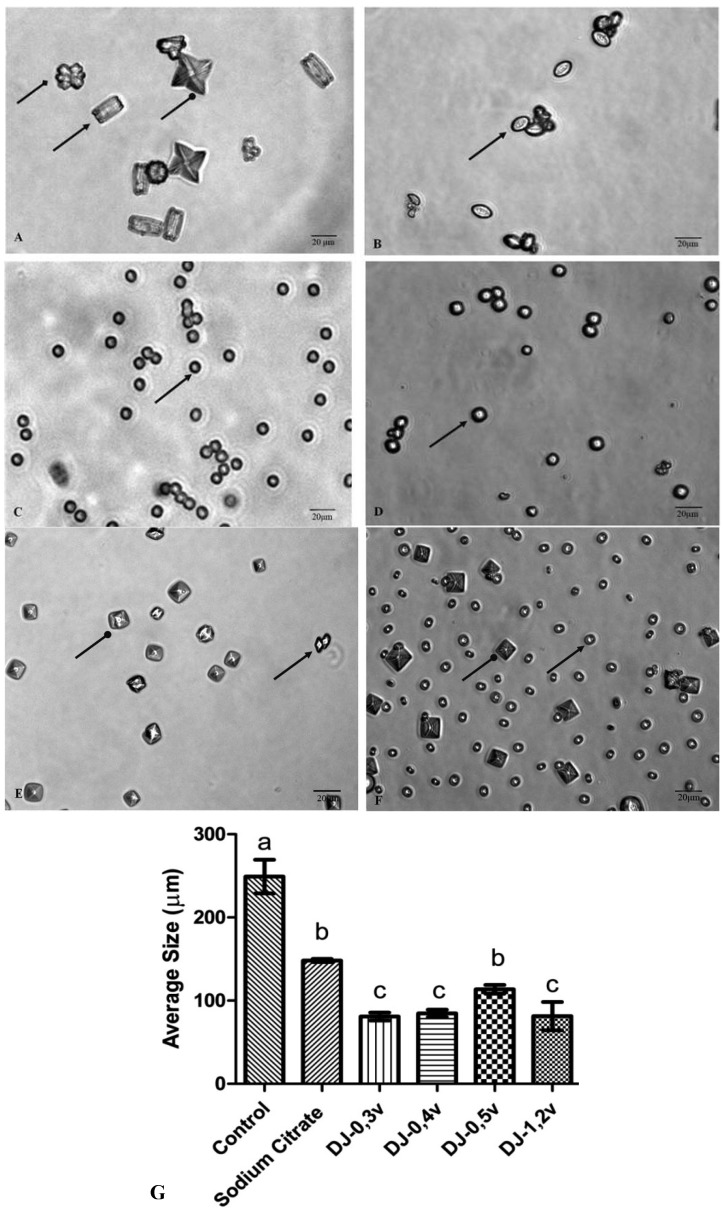
The CaOx crystals, observed under inversion ted microscope (60×) is formed in the meta stable solution of CaOx in the absence (**A**) and the presence of (**B**) Sodium Citrate (0.25 mM). (**C**) DJ-0.3v (0.1 mg/mL). (**D**) DJ-0.4v (0.1 mg/mL). (**E**) DJ-0.5v (0.1 mg/mL). (**F**) DJ-1.2v (0.1 mg/mL). 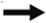 shows COM form; 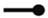 shows COD form and 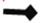 shows COT form; (**G**) Average size of formed crystals. Different letters (a,b,c) indicate a significant difference (*p* < 0.05) between the control, sodium citrate and the different polysaccharides. Scale Barr = 20 μm.

The crystals of calcium oxalate develop themselves in three different ways: monohydrated (COM), dihydrated (COD), and trihydrated (COT). The COM crystals have elongated tetragonal prism geometry, with an irregular outer surface, a dense structure, and high hardness. The COM stones basically consist of a nucleus where the crystals are deposited concentrically, and an intermediate radially striated layer [48]. The COD are crystals of calcium oxalate, with tetragonal bipyramid geometry, which are thermodynamically unstable. In contact with liquid, they gradually transform themselves into a more stable form, COM [49]. The COM form is found in large quantities in kidney stones, while COD is rarer. COT has a large thermodynamic instability, being seldom found within the stones. Figure 5A shows the crystals formed under control conditions. Under these conditions, three types of calcium oxalate crystals are formed, as described in the text of Figure 5. The observation of microscope slides in ten different fields has demonstrated that 68.5% are of type COM, 13.2% of type COD, and 18.3% are of type COT. The crystals formed in the presence of sodium citrate and polysaccharides are smaller (Figure 5) than those found in the control, in accordance to what was observed in the clustering assay, since all the samples inhibited the clustering of substantially similar shape.

The change in the morphology of the crystals as a result of the polysaccharides is quite visible. The more rounded geometry of crystals indicates that they are more amorphous, which is caused by the disruption of the crystal lattice due to the presence of polysaccharides that became associated with the crystals. Such geometry has a smaller surface area, when compared to COM crystals with sharp edges and points (control group), which facilitates the removal of these crystals from the body in urine [50].

Escobar and colleagues [46], working with negatively charged polymer [poly(ethyleneglycol)-block-poly (methacrylic acid) copolymer]—also known as PEG-b-PMAA—have demonstrated that it induces the formation of crystals at the expense of COD crystals. The authors suggest that PEG-b-PMAA, since it is hydrophilic, stabilizes COD crystals, preventing them from becoming COM. The two-glucans DJ-1.2v and especially DJ-0.5v are more readily soluble in water (data not shown) than DJ-0.3v and DJ-0.4v, and thus are more hydrophilic. These data lead us to propose that these glucans, as well as PEG-bPMAA, stabilize the COD crystals and prevent them from turning into COM.

The COD shape, although unstable, is very common in the urine of healthy patients, which indicates that the urine naturally contains molecules that stabilize the COD shape preventing its transformation into a COM geometric shape. This characteristic was also observed with glucans, especially in DJ-0.5v. Such stabilization has an efficient protective effect against urolithiasis since the crystals have a higher binding capacity to the cells of the renal tubule.

In order to verify the changes regarding the load related to the possible change in crystal structure after treatment with sulfated polysaccharides, the zeta potential of the samples was then carried out. The results are shown below.

### 2.5. Determination of Zeta Potential

The zeta potential (ζ) is a measure of the total charge of the particle surface (including molecules) in relation to the loads from the suspension liquid in which it is located. In the case of molecules such as polysaccharides, the ζ also reflects how much the conformation the molecule assumes can “expose” or “hide” the charges of the molecule. Thus, molecules that present different loads may have a similar ζ in one solution and different ζ in another type of solution.

In Table 3 we list the ζ values of the crystals formed from solutions under conditions similar to those in the calcium oxalate crystal inhibition assays. The average of the ζ in the crystals of untreated calcium oxalate was +8.39 ± 1.79 mV. The positive nature of the crystal surface can be correlated mainly with the presence of Ca^2+^ ions present within the crystal structure. All sulfated polysaccharides decreased the zeta potential of the calcium oxalate crystals. In the presence of the glucan DJ-0.5v, the ζ was +4.98 ± 1.1 mV. This polysaccharide was the one that least diminished the ζ of the crystals. This occurred due to the fact of DJ-0.5v was the less negatively charged among the four studied polysaccharides (Table 1). The sulfated polysaccharides DJ-0.3v and DJ-1.2v decreased the value of ζ of the crystals in similar amounts (4.5 ± 1.77 ± 0.9 and 4.19 mV, respectively). This may be related to the fact that the net charge of these fractions is similar (Table 1), as the zeta potential of these compounds. The fucan DJ-0.4v was the sulfated polysaccharide that most decreased the ζ crystals of CaOX, to which was given a value of 2.0 ± 0.45 mV. This effect was probably due to the greater amount of net negative charge of DJ-0.4v, compared to other sugars.

**Table 3:** Zeta Potential characteristics of crystals with treatment of sulfated polysaccharides from brown seaweed *Dictyopteris justii* at temperature 25 °C. a, b, c, d Different letters indicate a significant difference (*p* < 0.05). Zeta potential between each sample with CaOx. 1, 2, 3, 4 Different numbers indicate a significant difference (*p* < 0.05). Zeta potential between each sample without CaOx.

Samples	Zeta Potencial (mV)
**CaOx**	+8.39 ± 1.79 ^a^
**CaOx + Sodium Citrate**	+3.10 ± 1.11 ^b^
**CaOx + DJ-0.3v**	+4.5 ± 1.77 ^c^
**CaOx + DJ-0.4v**	+2.00 ± 0.45 ^d^
**CaOx + DJ-0.5v**	+4.98 ± 1.01 ^a^
**CaOx + DJ-1.2v**	+4.19 ± 0.9 ^e^

The results obtained from this study demonstrate that the sulfated polysaccharides of brown alga *D. justii* not only have a high ability to lower the crystallization of calcium oxalate, but also to act as antioxidants in different *in vitro* tests. Both of these activities acting together point out a promising possibility for the treatment of urolithiasis since it has been described that the reactive species are capable of causing renal injury, mainly caused by lipid peroxidation. At the same time, the oxalate crystals also promote an increase in reactive species, particularly in its formation, through excessive quantity of metal ions that can react with the hydrogen peroxide and increase the production of superoxide radicals and hydroxyl groups, further increasing renal injury [16]. Scavenging substances of free radicals such as sulfated polysaccharides have the effect of decreasing the deposition of crystals as well as repairing the tubular cell, promoting the return to homeostasis of the renal system.

## 3. Experimental

### 3.1. Extraction of Polysaccharides

The alga *Dictyopteris justii* (J.V.Lamouroux) was collected at Maracajaú Beach, Maxaranguape, Rio Grande do Norte (RN), Brazil. The seaweeds were identified by Valquiria P. Medeiros from the Federal University of Juiz de Fora, Juiz de Fora, MG, Brazil. The extraction of sulfated polysaccharides was adapted from the methodology described by Rocha *et al.* [20]. They were brought to the Laboratorio de Biotecnologia de Polimeros Naturais (BIOPOL), where they were cleaned with running water and oven dehydrated at 45 °C. They were subsequently crushed and subjected to a process of depigmentation and delipidation. After further drying under room temperature, two volumes of NaCl 0.25 M were added to the resulting mass while pH was adjusted to 8.0 with NaOH and Prozima (proteaze alkaline) added to the mixture for proteolytic digestion. After 18 h of incubation at 60 °C, the mixture was filtered and centrifuged (10,000 ×g, 10 min, 4 °C) and the supernatant was fractioned by precipitation with acetone. Thus, initially 0.3 volumes of acetone (4 °C) were added to the solution with gentle stirring and kept at 4 °C for 12 h. Then, the precipitate was separated from the solution by centrifugation (10,000 ×g, 10 min, 4 °C), dried, and stored for later analysis. To the supernatant obtained at this stage, more solvent was added until obtaining the turbidity of the material. This procedure was repeated with increasing amounts of acetone until no more turbidity was obtained from the supernatant.

### 3.2. Chemical Characterization, Monosaccharide Composition and Molecular Weight Determination

Total sugars were estimated by the reaction of phenol-H_2_SO_4_ using L-fucose for fucans and D-glucose for glucans as standards, as described by Dubois *et al.* [51]. The fucans were hydrolyzed (4 M HCl, 100 °C, 6 h) and the sulfate content was determined according to the barium-gelatin method [52] by using a standard curve of sodium sulfate. The protein content, in turn, was measured by using the modified Bradford method [53], with bovine serum albumin as standard. The monosaccharide composition was determined as described in Rocha and colleagues [20]. As a standard, we used the sugars: glucose, xylose, galactose, mannose, glucuronic acid, rhamnose, fucose, and arabinose.

In order to estimate the molecular weight of the polysaccharides, they were subjected to gel permeation chromatography on Sephadex G-100 (140 × 1 cm) using 0.2 M acetic acid/0.15 M NaCl as an eluent. The elution was monitored for total sugar [51]. Dextrans of different molecular weights were used as standards.

### 3.3. Fourier Transformed Infrared Spectroscopy (FT-IR)

The sulfated polysaccharides (5 mg) were thoroughly mixed with dry potassium bromide. The infrared spectra between 500 and 4,000 cm^−1^ were performed with a KBr tablet and the polysaccharide and they were measured with a Thermo Nicolet Nexus 470 ESP FT-IR spectrometer. Thirty-two scans at a resolution of 4 cm^−1^ were calculated and referenced against air.

### 3.4. Antioxidant Activity

#### 3.4.1. Determination of Total Antioxidant Capacity

This assay is based on the reduction of Mo (VI) Mo (V) by sulfated polysaccharides and subsequent formation of a phosphate green complex/Mo (V) with acid pH. Tubes containing sulphated polysaccharides and reagent solution (0.6 M sulfuric acid, 28 mM sodium phosphate, and 4 mM ammonium molybdate) were incubated at 95 °C for 90 min. After the mixture had cooled to room temperature, the absorbance of each solution was measured at 695 nm against a blank. Total antioxidant capacity was expressed as ascorbic acid equivalent.

#### 3.4.2. Reducing Power

The reducing power was quantified according to the methodology described by Wang *et al*. [54]. The test samples (4 mL) in different concentrations (0.1–1.0 mg/mL) was mixed in a phosphate buffer (0.2 M, pH 6.6) with potassium ferricyanide (1%) and incubated for 20 min at 50 °C. The reaction was interrupted by the addition of TCA (trichloroacetic acid) to 10%. Subsequently, distilled water and ferric chloride (0.1%) were added to the samples. Readings were taken at 700 nm.

#### 3.4.3. Hydroxyl Radical Scavenging Activity Assay

The scavenging assay of the hydroxyl radical was based on the Fenton reaction (Fe^2+^ + H_2_O_2_ → Fe^3+^ + OH^−^ + OH). The results were expressed as inhibition rates. The hydroxyl radicals were generated using 3 mL of sodium phosphate buffer (150 mM, pH 7.4) containing 10 mM FeSO_4_・7H_2_O, 10 mM EDTA, 2 mM of sodium salicylate 30% H_2_O_2_ (200 mL), and different concentrations of SP. In the control, sodium phosphate buffer replaced H_2_O_2_. The solutions were incubated at 37 °C for 1 h, and the presence of the hydroxyl radical was detected through the monitoring of the absorbance at 510 nm. Gallic acid was used as a positive control.

#### 3.4.4. Superoxide Radical Scavenging Activity Assay

This assay was based on the ability of SPs to inhibit the photochemical reduction of tetrazolium nitroblue (NBT) in the riboflavin-light-NBT system. Every 3 mL of reaction mixture contained 50 mM of phosphate buffer (pH 7.8), 13 mM of methionine, riboflavin 2 mM, EDTA at 100 mM, NBT (75 mM), and 1 mL of the sample solution. After 10 min of illumination with a fluorescent lamp for the production of blue formazan to occur, the samples were read at 560 nm. Identical tubes of the reaction mixture were kept in the dark and served as blanks for the reaction. Gallic acid was used as a positive control.

#### 3.4.5. Ferrous Chelating

The capacity to chelate iron from the ionic samples was investigated using the following methodology: SP at different concentrations was added to a reaction mixture containing FeCl_2_ (0.05 mL, 2 mM) and ferrozine (0.2 mL, 5 mM). The mixture was stirred and incubated for 10 min at room temperature and the absorbance of the mixture was measured at 562 nm against a blank. EDTA was used as positive control.

#### 3.4.6. Copper Chelating

The ability to chelate the copper ion from the extracts was determined by the method described by Anton [55]. Pyrocatechol violet, the reagent used in this assay, has the ability to associate with certain cations such as aluminum, copper, bismuth, and thorium. In the presence of chelating agents this combination is not formed, resulting in decreased staining. This reduction thus allows estimating the chelating activity of the copper ion from the fraction from *Dictyopteris justii*. The test is performed in 96-well microplates with a reaction mixture containing different concentrations of samples (0.1–20 mg/mL), pyrocatechol violet (4 mM), and copper II sulfate pentahydrate (50 mg/mL). All wells were homogenized with the aid of a micropipette and the solution absorbance was measured at 632 nm. The ability of the samples in chelating the copper ion was calculated using the following equation:

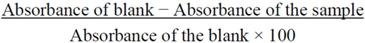


### 3.5. Calcium Oxalate Crystallization Assay

The effect of SP in the crystallization of calcium oxalate was spectrophotometrically measured for 30 min at 620 nm, as described by Zhang *et al*. [17]. This assay is based on quantification by optical density of metastable solutions of Ca^2+^ and Ox, by means of a mixture of calcium chloride (8 mmol/L) and sodium oxalate (1 mmol/L), 200 mmol/L of sodium chloride, and 10 mmol/L of sodium acetate. The concentrations of compounds present in this mixture are close to the physiological urinary concentrations. The CaCl_2_ (1.0 mmol/L) solution was constantly stirred at 37 °C either in the absence or the presence of different concentrations of the sulfated polysaccharides or sodium citrate (0.25 mM) as a positive control. After obtaining a stable baseline, crystallization was induced by the addition of a solution of Na_2_C_2_O_4_ (1.0 mmol/L) to achieve final concentrations of 4 mmol/L of calcium and 0.5 mmol/L of oxalate. By beginning with a linear regression analysis, it was possible to measure the percentage of crystallization inhibition. The referring percentage was calculated from the rate of nucleation and aggregation as follows: [1 − (SN_A_/SN_C_)] × 100 for the percentage of nucleation, having SN_A_ as the inclination of the absorbance of the salt solution in the presence of the samples and NS_C_ the inclination of the control; [1 − (SA_A_/SA_C_)] × 100 for the percentage of aggregation where SA_A_ is the inclination of the absorbance of the solution in the salts in the presence of samples and, SA_C_, the inclination of the absorbance of the control.

### 3.6. Image Analysis Crystal Morphology

The crystals were induced to take shape in the presence or the absence of SP or sodium citrate 0.25 mM. After 30 min, the solutions were centrifuged (5000 × g) and the supernatant was discarded. The crystals were then suspended in 0.5 mL of water and a part of 0.1 mL was put on a histological blade and taken to a microscope. The crystal morphology was analyzed in 10 randomly selected fields at 60 × magnification. Images were captured from different fields. We performed three different experiments.

### 3.7. Zeta Potential (ζ) Measurements

The crystals were induced to form in the presence or absence of SP or sodium citrate 0.25 mM. After 30 min the solutions were centrifuged (5000 ×g). The crystal concentrate was then suspended in 1.5 mL of water, and the zeta potential of the ζ samples was obtained using a Zeta Plus^®^ analyzer.

## 4. Conclusions

The brown alga *Dictyopteris justii* synthesizes four populations of sulfated polysaccharides DJ-0.3v (glucofucoxyloglucuronan), DJ-0.4v (heterofucan), DJ-0.5v (sulfated glucan), and DJ-1.2v (sulfated glucan). All fractions presented antioxidant activity, with the fucan DJ-0.4v standing out as the most potent in all of the assays. Fucan DJ-0.4v was also the fraction with the better capacity to inhibit the crystallization of calcium oxalate. Also noteworthy is the fraction DJ-0.5v that, besides inhibiting, was also capable of stabilizing the COD crystals, preventing them from turning into COM crystals. These polysaccharides are, therefore, promising agents for possible application in the treatment of urolithiasis.
